# Some Practical Observations on Inflammations of the Palmar Surface of the Hand

**Published:** 1890-03

**Authors:** Geo. H. Winston

**Affiliations:** West Point, Ga.


### Southern Medical Record.

A MONTHLY JOURNAL OF MEDICINE AND SURGERY.

VOL. XX. ATLANTA, GA., MARCH, 1890. NO. 3.

### Original Articles.

### SOME PRACTICAL OBSERVATIONS ON INFLAMMATIONS OF THE PALMAR SURFACE OF THE HAND.

BY GEO. H. WINSTON, M. D., WEST POINT, GA.

The hand has been considered, by some, and more especially ancient philosophers, as constituting the true superiority which man enjoys above the brute. Certainly it is mind's most efficient servant, and represents the most nearly perfected type of animal mechanism. A permanent injury to this member means a permanent injury to the economy's usefulness. And yet it is peculiarly liable to injury, infection and phlegmonous inflammations, and, owing to its anatomical idiosyncrasies, especially susceptible to permanent impairment of functions from these processes. The hand is our agent in nearly every act, and must necessarily meet with much sceptic matter as well as bear many lesions. And once injured or infected, it is far more liable than other portions of the body to take on a powerful and rapidly extending inflammatory action. This is due to the peculiar arrangement of the connective tissue packing of the palmar surface. The connective tissue of the dorsum is loose and has the usual arrangement of subcutaneous areolar tissue. That of the palmar surface, however, is composed of rather dense bundles of fibres, which have a general direction extending from, and closely attached to, the cutaneous surface inward and reaching the deeper structures of the hand. This is more especially true of the arrangement in the fingers. The lymphatic channels take the same general direction as the connective tissue fibres. With such an arrangement, allow some finger to be injured, or say inoculated through a slight and perhaps unnoticed abrasion of the cuticle. In a few hours an active inflammatory process is developed. The consequent exudation and swelling cannot well extend laterually on account of the peculiar arrangement of the connective tissue fibres. The closely attached integument prevents any escape outward. The original cutaneous lesion is, if not already closed, too small to afford any relief. Even if a free incision originally existed, it has been, perhaps, hermetically sealed by that omnipresent—and, for evil, omnipotent—demon of domestic surgery, "sticking-plaster." Inward becomes virtually the line of least resistence. If infection exists, the capillary lymph channels are present to rapidly convey the poison in the same direction. By that tension, so easily induced by the condition of things above described, new inflammatory products are induced; more tension created; destructive processes rapidly occur; pus forms: and the whole process rapidly extends inward toward the bone and tendon sheaths. When the morbid action reaches the tendons, it shows a disposition to rapidly extend along them, leaving in its train a hand rendered useless by adhesions. The bone, owing to the inflammatory action taken on by the periostium, and later by its own structrue, is liable to necrosis. This liability is doubly increased by the condition of the soft parts, above described. But the destruction of phalanges and tendons rendered useless by adhesions, are not the only dangers to be anticipated. The process and products of inflammation, being as it were imprisoned from without, and being aided by the peculiar arrangement of the tendon bursæ, show a decided tendency to extend along them toward and reaching the wrist joint, and even to invade high up the arm. In the more severe cases the presence of the imprisoned pus lays the patient liable to the too often disastrous results of pyaemia. One case, at least, has come to the author's notice, in which death resulted from this cause. Stiff joints, rotting bones, amputated hands, even death itself are the results that bear witness, with not always silent reproach, against him who would treat inflammations of the palmar surface of tne hand as matters trivial.

How may we avoid such unpleasant witnesses? My plan of treatment can be enunciated in a few words:—relieve tension and give free passage for the escape of inflammatory products, which practically means incision. When a hand is wounded —more especially if it be a punctured wound or with suspicion of infection—no attempts should be made to close it except such as will give free drainage from its bottom. If it be deep and its external opening be quite small, the cutaneous lesion may be occasionally enlarged to advantage:—at all events the opening should be kept patulous and an attempt be made to secure healing from the bottom. A clean cut, uninfected, incised wound may be, after an attempt to render it aseptic, closed. But it should be carefully watched for signs of the development of any considerable amount of active inflammation.

When such a process has been aroused and the parts begin to show the development of any great amount of tension, it is my custom to lay them open. I know that it is the judgment of the majority of the profession to await the formation of some considerable amount of pus. But, owing to the anatomical peculiarities of this region, the presence of an active inflammation is only too liable to lead to the quick formation of great tension and the rapid destruction of the interior structures. Even at the early date at which I open such inflamed parts, a careful examination will often show the presence of a few drops of pus: thus proving that the destruction of tissue has begun. By relieving tension, we will prevent any great amount of destruction of tissue.

Now, in relieving tension and fully draining the part, we use the best possible means to prevent the spread of the inflammation and to cut short the pathological process.

CASE:—Gus. W., black. aged 35 years. Presents syphilitic diathesis, of which no history could be obtained. History of tendency to the development of dactylites upon slight traumatisms. These attacks had previously been allowed to run on, with great suffering to the patient, to the formation of considerable amount of pus before they were opened. He presented himself to me on August 5th, 1889, his right ring finger exhibiting a violent and tightly swollen inflammation, completely circumscribing the second phalanx. I could detect no fluctuation, despite which I made a free incision on the palmar surface, dividing the tissues down to the bone. Not a drop of pus escaped. Laid a piece of iodoform gauze in wound, put on light moist dressing. Saw the case again August 11th, and found the finger practically well. The patient had had literally no pain after the incision, and, to use his own words, I "had saved him from at least ten days of pain."

I would not be considered as denying here the utility of other methods of relieving inflammatory action. But I would emphasize the fact that, when a certain amount of tension is reached, incision is the surest, and often the only method of checking the process.

When pus has formed, incision becomes almost the only procedure. As before intimated, the sooner this is done the better. Owing to the anatomical peculiarities just mentioned, the tendency of pus is not toward the surface, but rather inward toward the tendon sheaths and periosteum. If the surgeon does not open the forming abscess and favor the escape of pus, he may with tolerable certainty reckon upon its extending inward upon its errand of mischief.

Cataplasms are in this region worse than useless. Too often used in other situations to hide ignorance or indecision and to keep up the appearance of "doing something," they here become criminal. They are favoring the formation of a suppuration whose tendency is to extend inward. and hence incalculably increase the trouble instead of hastening its alleviation. I have now in mind a case of a lady whose hand was bitten by a spider. Her physician ordered cataplasms, which he persisted in using. This treatment resulted in the formation of a horrible palmar abscess, with minor sacs of pus extending up the fore-arm. The life of the patient was saved by the advent of another physician, who made free openings. but the hand is useless to this day. Here is a case where cataplasms ruined a hand. Had the process been freely opened upon the first sign of pus, this deformity, as well as many days of anxiety and pain, would have been avoided.

The abscess being freely incised, care should be taken to prevent its premature closure. A piece of iodoform gauze may be placed in the opening; or, if this be not at hand, some absorbent cotton that has been squeezed out in an antiseptic solution, may be used instead. The abscess should be dressed every day and thoroughly washed out with a carbolic acid solution, 1 to 20. In examining a hand much swollen by a phlegmon, care should be taken not to be deceived by appearances. The skin of the palmar surface, being closely attached, may not show much swelling, while the loose subcutaneous areolar tissue of the dorsum may become enormously infiltrated. Thus one might be led into opening the back of the hand, when the trouble lies entirely upon the other side. In choosing a point for incision, I usually select that point where the pus lies nearest the surface. If the palmar arch be cut, it may be readily tied, though this accident should, if possible, be avoided. This may be done, as a rule, by avoiding that space which lies within the W. That is more or less perfectly traced upon the palm.

It is a point of interest to note that abscess of the thumb or little finger is more liable to extend to the palm than when upon the other digits. This is due to the fact that the tendon sheaths of the thumb and little finger are continuous with the general tendon bursa of the palm, while those of the other fingers are separate. For a dressing I use any of the light, moist kind, preventing its drying, when necessary, by covering with rubber tissue. This dressing will better absorb the discharges, and prevents them drying and becoming hard.

In conclusion, I would emphasize the fact that this is a situation above all others, where early and prompt treatment of phlegmon is necessary, and that, in the vast majority of cases, immediate incision is the proper procedure.

